# Constitutive gene expression profile segregates toxicity in locally advanced breast cancer patients treated with high-dose hyperfractionated radical radiotherapy

**DOI:** 10.1186/1748-717X-4-17

**Published:** 2009-06-04

**Authors:** Luis Alberto Henríquez Hernández, Pedro Carlos Lara, Beatriz Pinar, Elisa Bordón, Carlos Rodríguez Gallego, Cristina Bilbao, Leandro Fernández Pérez, Amílcar Flores Morales

**Affiliations:** 1 Canary Foundation of Investigation and Health (FUNCIS), Spain; 2 Canary Institute for Cancer Research (ICIC), Spain; 3 Clinic Sciences Department of Las Palmas de Gran Canaria University (ULPGC), Spain; 4 Radiation Oncology Department, Hospital Universitario de Gran Canaria Dr Negrín, Spain; 5 Inmunology Department, Hospital Universitario de Gran Canaria Dr Negrín, Spain; 6 Molecular Endocrinology Group, Center for Molecular Medicine, Karolinska Intitute, Stockholm, Sweden

## Abstract

Breast cancer patients show a wide variation in normal tissue reactions after radiotherapy. The individual sensitivity to x-rays limits the efficiency of the therapy. Prediction of individual sensitivity to radiotherapy could help to select the radiation protocol and to improve treatment results. The aim of this study was to assess the relationship between gene expression profiles of *ex vivo *un-irradiated and irradiated lymphocytes and the development of toxicity due to high-dose hyperfractionated radiotherapy in patients with locally advanced breast cancer. Raw data from microarray experiments were uploaded to the Gene Expression Omnibus Database  (GEO accession GSE15341). We obtained a small group of 81 genes significantly regulated by radiotherapy, lumped in 50 relevant pathways. Using ANOVA and t-test statistical tools we found 20 and 26 constitutive genes (0 Gy) that segregate patients with and without acute and late toxicity, respectively. Non-supervised hierarchical clustering was used for the visualization of results. Six and 9 pathways were significantly regulated respectively. Concerning to irradiated lymphocytes (2 Gy), we founded 29 genes that separate patients with acute toxicity and without it. Those genes were gathered in 4 significant pathways. We could not identify a set of genes that segregates patients with and without late toxicity. In conclusion, we have found an association between the constitutive gene expression profile of peripheral blood lymphocytes and the development of acute and late toxicity in consecutive, unselected patients. These observations suggest the possibility of predicting normal tissue response to irradiation in high-dose non-conventional radiation therapy regimens. Prospective studies with higher number of patients are needed to validate these preliminary results.

## Introduction

Radiation is an effective therapy in patients with local advanced breast cancer (LABC) [1,2]. Tumor control by radiotherapy (RT) requires the use of maximum dose that can be delivered while maintaining a tolerance risk of normal tissue toxicity [3]. Better local control outcomes with an acceptable toxicity have been obtained by using high total doses radiation administered in two small fractions per day compared with standard RT protocols [4]. Some patients treated with RT will develop early or late reactions limiting the efficacy of RT. Knowledge of individual variations of normal tissue toxicities determining tolerance would be of great value in patients treated with high-dose radiation protocol [5]. Microarray technology is a high throughput method that allows large scale genomic studies. Because intrinsic radiosensitivity is genetically determined, different cells from the patient can be used to measure sensitivity to radiation [6]. Few studies have been published with regard to radiation induced toxicity and microarrays [2,7-10]. Patients were previously selected according to the clinical toxicity observed and only three publications included breast cancer patients [see Additional file 1].

The aim of this study was to assess the relation of the gene expression profile from un-irradiated and irradiated lymphocytes and the development of toxicity due to RT in patients with LABC.

## Patients and methods

**Figure 1:**
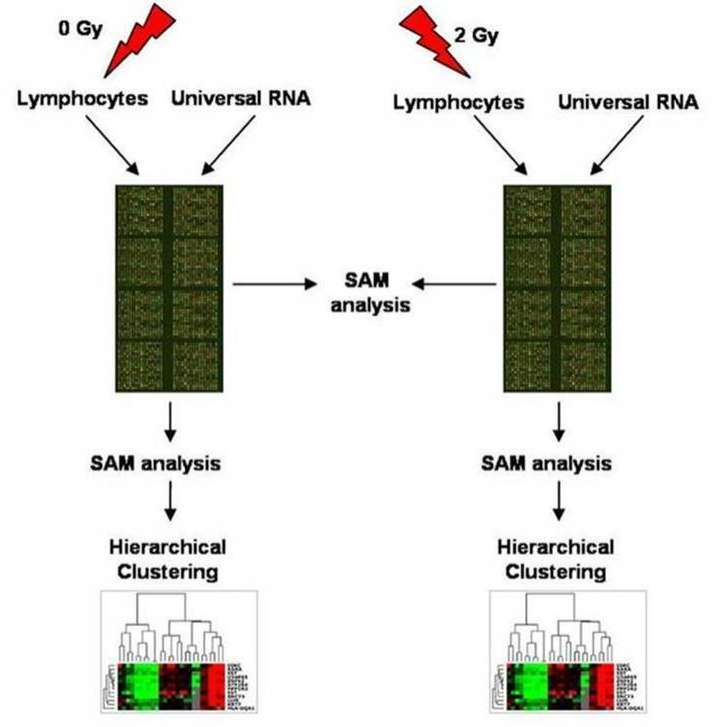
**Experimental design**. RNA from lymphocytes treated with 0 and 2 Gy dose of radiation were compared against a human universal RNA. SAM analyses were performed to disclose significant regulated genes in these two ways. In order to explore genes modulated by radiation, a two-class paired test was performed using SAM. To discriminate genes that could be significantly associated with RT toxicity, non-supervised hierarchical clustering, in MeV, was used to visualize the whole set of significant genes modulated before and after X-ray exposure in patients with and without acute/late toxicity.

Twelve consecutive patients treated between 1991 and 1997 by a hyperfractionated dose-escalation radiation therapy schedule at the Hospital Dr. Negrín suffering from LABC were prospectively recruited and inform consent was given. The study was approved by the Research and Ethics Committee of our institution. Blood samples were extracted and tested during 2005 and follow up was closed on December 2008. Characteristics of the patients are shown in Table 1. Early toxicity was evaluated during and at the end of RT, and late toxicity was evaluated at 6-month follow-up examination. The RTOG morbidity score system was used to classify the toxicity of patients into three levels: grades 1, 2 and 3–4 (Table 2). All patients were referred to recieve 60 Gy to the whole breast over a period of 5 weeks in two daily fractions of 1.2 Gy separated by at least 6 h on 5 days each week, and followed by a boost of 21.6 Gy to a total dose of 81.6 Gy. Culture of lymphocytes and radiation protocol details were previously reported [11]. Twenty four independent hybridizations were performed to compare lymphocytes from twelve patients, before and after 2 Gy irradiation, against a human RNA universal control. A microarray containing 35.327 human 70-mer oligo probe sets, produced at the SweGene DNA Microarray Resource Center (Lund University, Sweden) was used. Array scanning, image analysis and data normalization were performed as previously described [12,13]. Identification of differentially-expressed genes was performed using the SAM (Significance Analysis for Microarrays) statistical technique [14]. A *q *value was assigned for each of the detectable genes in the array measuring the lowest false discovery rate (FDR). Genes with a FDR of less than 10% were considered to present significant differential expression. Thus, we studied gene expression profile of lymphocytes treated with 0 and 2 Gy separately. To explore genes modulated by radiation, we also compared gene expression profiles of lymphocytes treated with 0 versus 2 Gy. T-test and ANOVA test [15,16] were used to compare the set of genes significantly regulated, in un-irradiated and in 2 Gy-irradiated lymphocytes, with toxicity. Non-supervised hierarchical clustering [17] was made using MultiExperiment Viewer (The Institute for Genomic Research, ). A genetic signature that could separate toxicity and non toxicity in a constitutive and in a modulated-by-radiation way was performed (Figure 1). Functional classification and pathway analysis of expressed genes were performed by using the web-based tools Onto-Express (OE) and Pathway-Express (PE) (Intelligence Systems and Bioinformatics Laboratory, Wayne University, Detroit, MI. ) [18,19]. OE classify genes in order to biological process (BP), cellular component and molecular function, and it is able to estimate statistical differences between different gene ontology terms [20]. PE is based on a novel method that uses a system biology approach that includes important biological factors that describes how these genes interacts and the type of signaling interactions between them [21,22].

**Table 1:** Characteristics of the patients included in the study. Age, menopause status, characteristics of the tumor and systemic treatment were added.

***Characteristic***	***n***	***%***
Age		
<60 years	5	41.7
≥ 60 years	7	58.3
Menopause		
Premenopausal	3	25.0
Postmenopausal	9	75.0
Tumor type		
Inflammatory	5	41.7
Non-inflammatory	7	58.3
Tumor size (T)		
T3	1	8.3
T4a	1	8.3
T4b	5	41.7
T4c	0	0
T4d	5	41.7
Nodes (N)		
N0	7	58.3
N1	3	25.0
N2	2	16.7
Metastasis (M)		
M0	12	100
M1	0	0
Systemic treatment		
Chemotherapy	1	8.3
Hormonal therapy	1	8.3
Chemotherapy-hormonal therapy	10	83.4

**Table 2:** Grade of acute and late toxicity of patients included in the study.

	***Patient Code***	***Age***	***Acute Toxicity***	***Late Toxicity***
***1***	01	34	Grade 1	Grade 2
***2***	02	60	Grade 1	Grade 3
***3***	03	47	Grade 1	Grade 2
***4***	04	68	Grade 2	Grade 2
***5***	05	67	Grade 2	Grade 3
***6***	06	70	Grade 2	Grade 2
***7***	07	63	Grade 2	Grade 3
***8***	08	72	Grade 2	Grade 3
***9***	09	57	Grade 3	Grade 3
***10***	10	64	Grade 3	Grade 3
***11***	11	47	Grade 3	Grade 2
***12***	12	53	Grade 4	Grade 4

Age is shown.

## Results

Comparison of gene expression profiles from 0 Gy and 2 Gy-treated lymphocytes, using two-class paired test in SAM program, identified a total of 81 genes significantly regulated by RT [see Additional file 2]. We could not cluster these genes in order to segregate patients with acute or late toxicity. PE was used to explore biological pathways significantly regulated by radiation. Fifty seven genes were mapped and PE identified 50 pathways significantly regulated (p < 0.01). Among the RT modulated pathways there were cell cycle, nucleotide excision repair, DNA replication, mismatch repair; MAPK, erbB, and VEGF signaling, ubiquitination mediated proteolysis, notch and Wnt [see Additional file 3]. A functional classification of 81 regulated genes was made using OE. Forty-five genes were classified according to the BP and several processes were modified by RT [see Additional file 4].

**Figure 2:**
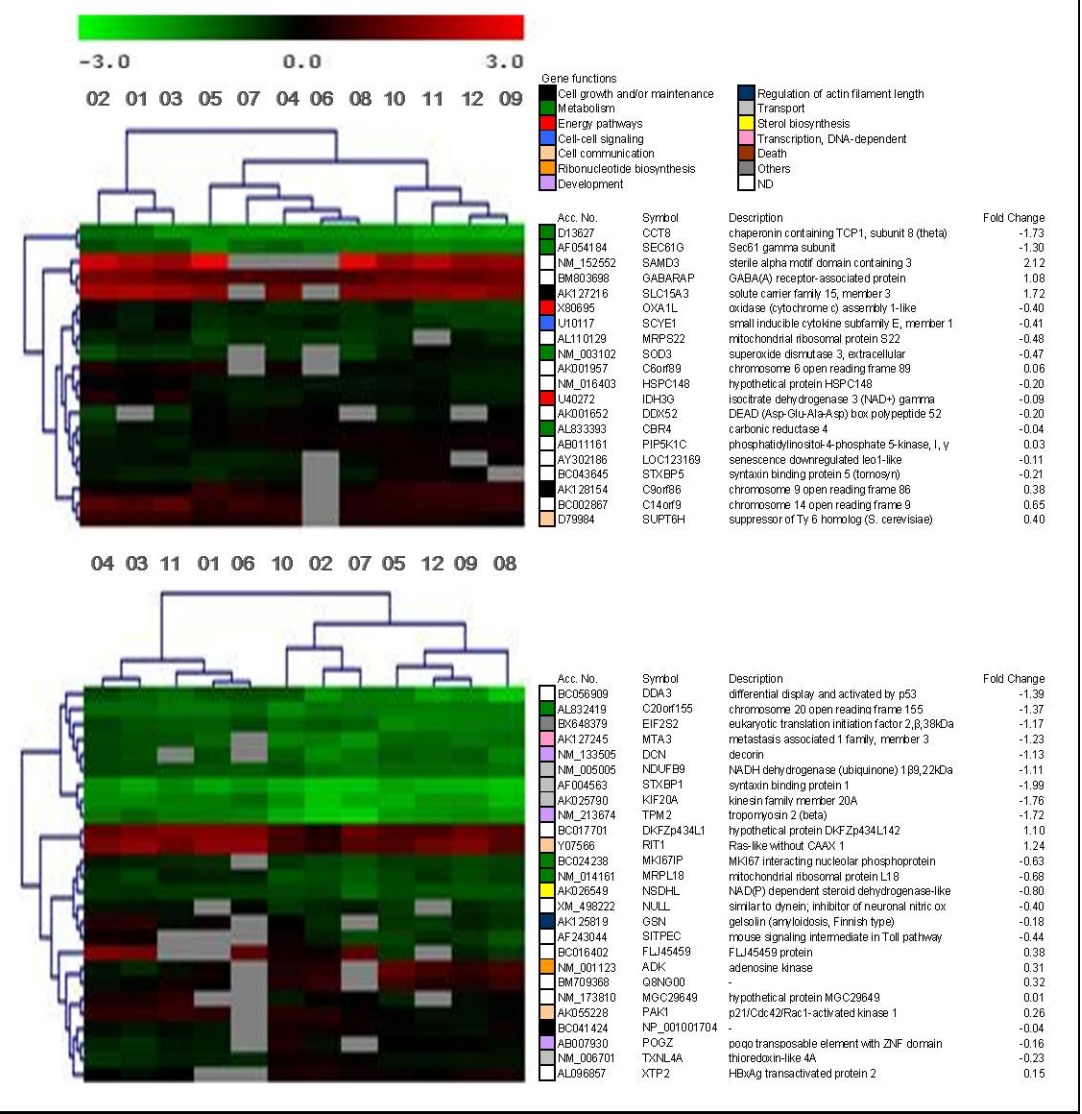
**Non-supervised hierarchical clustering of constituve genes regulated in un-irradiated lymphocytes**. Clustering used Euclidean distance correlation and average linkage, and was processed and displayed with MultiExperiment Viewer . Upper panel shows a 20 gene set that segregated patients with different grade of acute toxicity (First three patients, grade 1; next five patients, grade 2; last four patients, grades 3–4) ANOVA test, p < 0.01. Lower panel shows a 26 gene set that segregated patients with different grade of late toxicity (First five patients, grade 2; last seven patients, grades 3–4) T-test, p < 0.01. The dendogram to the left of the heat map shows clustering of the genes. Accession number, gene symbol, gene description and fold change were added. Colour boxes indicate the biological process of each gene.

SAM analysis from un-irradiated lymphocytes revealed 7391 constitutive regulated genes. ANOVA test in MeV identified 20 genes that segregated patients with grade 1, from grade 2 and grade 3–4 acute toxicity (p < 0.01) (Figure 2). PE identified 6 pathways significantly regulated (p < 0.01): protein export, regulation of autophagy, vibrio cholerae infection, phosphatidylinositol signaling system, focal adhesion and regulation of actin cytoskeleton [see Additional file 5]. OE classified 14 genes according to the BP. Processes as chromatin remodeling, regulation of endothelial cell proliferation, oxidation reduction and cellular respiration were constitutively modulated (p < 0.05) [see Additional file 6]. The same strategy was followed for late toxicity. T-test identified 26 genes that constitutively segregated patients who suffered severe late toxicity from patients who did not (p < 0.01) (Figure 2). PE identified 9 pathways significantly regulated (p < 0.01): regulation of actin cytoskeleton, MAPK signaling, epithelial cell signaling in helicobacter, Erb B signaling pathway, renal cell carcinoma, natural killer cell mediated cytotoxicity, T cell receptor signaling, axon quidance and focal adhesion [see Additional file 5]. The role of PAK1 (p21-Cdc42/Rac)-activated kinase 1 must be highlighted since it was involved in all the 9 pathways. OE scored 13 genes according to the BP. Processes significantly regulated (p < 0.05) were: lipid, cholesterol and sterol-biosynthetic processes, cytoskeleton organization and biogenesis, positive regulation of gene specific transcription, hair follicle development, ER-nuclear signaling pathway, positive regulation of JNK activity and others [see Additional file 7].

**Figure 3:**
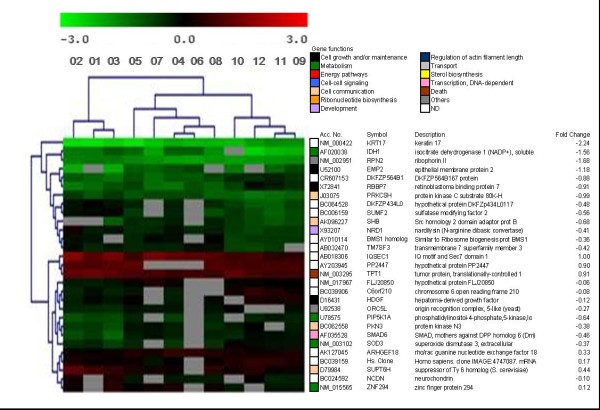
**Non-supervised hierarchical clustering of genes regulated in irradiated lymphocytes (2 Gy)**. Clustering used Euclidean distance correlation and average linkage, and was processed and displayed with MultiExperiment Viewer . A 29 gene set segregated patients with different grades of acute toxicity (First three patients, grade 1; next five patients, grade 2; last four patients, grades 3–4) ANOVA test, p < 0.01. The dendogram to the left of the heat map shows clustering of the genes. Accession number, gene symbol, gene description and fold change were added. Colour boxes indicate the biological process of each gene.

Lymphocytes from patients were also irradiated at 2 Gy dose. SAM identified 7393 genes significantly regulated. ANOVA test (p < 0.01) identified 29 genes that separated patients with grade 1, from grade 2 and grade 3–4 acute toxicity (Figure 3). We did not observe common genes between this set of genes and those corresponding to un-irradiated lymphocytes (constitutive genes). PE identified 4 significantly regulated pathways (p < 0.01): phosphatidylinositol signaling system, regulation of actin cytoskeleton, cell cycle and TGF-beta signaling pathway [see Additional file 5]. OE scored 15 genes according to BP with some processes also significantly regulated [see Additional file 8]. We could not obtain a consistent set of genes able to separate patients with regard to late toxicity in irradiated lymphocytes (Table 3).

**Table 3:** Summary of results obtained after non-supervised hierarchical clustering.

***Group***	***Association***	***Gene Set***	***N° of pathways***
***Acute 0 Gy***	Y	20	6
***Late 0 Gy***	Y	26	9
***Acute 2 Gy***	Y	29	4
***Late 2 Gy***	N	11	-

"Y" indicates positive association between toxicity and gene expression. "N" indicates no association. The number of genes associated for each group were included, as well as the number of significant pathways derived from the gene sets.

## Discussion

Constitutive gene expression pattern from un-irradiated lymphocytes can segregates LABC patients with acute and late toxicity from patients without toxicity after hyperfractionated radiation therapy treatment. Using 2 Gy irradiated lymphocytes from the same patients we could only observe association related to acute toxicity. Few series were published to explore the relation of radiation induced toxicity and microarray, and only three were referred to breast cancer [7,9,10]. The paper published by Svensson et al. is similar to the present work in relation to the experimental design, but was assessed in prostate cancer patients [2]. Recently, Rødningen et al. published two relevant papers [10,23]. Our results were not similar related to genes involved in late toxicity. Anyhow, we coincided in relation to some BP. Differences in cell type, microarray platform, experimental design, RT protocol and statistical strategy could explain those differences. Compared with previously available studies, this is the first work in which: i) patients were consecutive and non-previously selected, ii) patients were treated with high-dose radiation protocol with altered fractionation, iii) the complete human genome was analyzed and iv) comparative studies of constitutive gene expression profiles of LABC patients and toxicity were made.

Pak1 seems to have an important role in late toxicity in our study. Pak1 overexpression is related to apoptosis-resistance in normal and tumour cells [24]. An appropriate apoptotic response seems to protect normal tissue against radiation late toxicity [25]. Therefore, over-expression of Pak1 observed in our patients would be related to resistance to late toxicity. The role of PAK1 in late toxicity should be explored.

This long term study makes a novel contribution to shed light to the relationship between the constitutive gene expression profile of peripheral blood lymphocytes and toxicity after RT. This analysis opens the possibility that the different constitutive expression levels of a selected group of genes would predict acute and late toxicity caused by RT. The feasibility and cost effectiveness of this assay would encourage clinical application in larger series of patients. Further prospective experiments are needed to validate those genomic profiles.

## Abbreviations

LABC: Local Advanced Breast Cancer; BP: Biological Process; FDR: False Discovery Rate; MeV: Multiexperiment Viewer; OE: Onto-Express; PE: Pathway-Express; RT: Radiotherapy; SAM: Significant Analysis for Microarray.

## Competing interests

The authors declare that they have no competing interests.

## Authors' contributions

LAHH has made the microarray analysis as well as the interpretation of the data, likewise the writing of the manuscript and the confection of tables and figures.

PCL has been involved in conception and design of the study as well as in drafting the manuscript, and has given final approval of the version to be published.

BP has made the selection of patients, the evaluation of clinical variables and grade of toxicity as well as all the aspects related with the patients selected.

EB and CRG have made the irradiation experiments with lymphocytes and the obtaining of samples.

CB and LFP have been involved in revising the manuscript critically for important intellectual content.

AFM has made the microchip experiments, sample preparation, images acquisition and initial processed of data.

## Supplementary Material

Additional file 1 **Studies that have applied microarray analysis to compare gene expression profiles in patients with severe versus mild normal tissue damage after radiotherapy**. Brief summary of studies related to radiotherapy and microarrays. The table includes the author's name and the year of publication, the cell type used the tumour type, some characteristics of the study and the most relevant findings. Click here for file

Additional file 2 **Genes significantly regulated by radiotherapy in human lymphocytes**. Eighty one genes regulated by radiation. The table contains gene symbol, description, numerator, fold change, q value, gene id, transcript id, RefSeq, description RefSeq and GeneBank Acc number. Click here for file

Additional file 3 **Pathways significantly regulated by radiotherapy in human lymphocytes**. Fifty pathways regulated by radiation. The table contains rank, database name, pathway name, impact factor, genes in pathway, input genes in pathway, pathway genes on chip and p value. Click here for file

Additional file 4 **Functional Classification. Genes modulated by radiotherapy**. The table contains the functional classification in relation to biological process of 81 genes modulated by radiotherapy. Click here for file

Additional file 5 **Canonical pathways that were significantly modulated in the different set of genes**. Pathways modulated and related to acute and late toxicity, 0 and 2 Gy. Pathway name, p-value, gene name and GeneBank accession number were included. Click here for file

Additional file 6 **Functional Classification.** Acute toxicity, 0 Gy. The table contains the functional classification in relation to biological process of genes regulated in un-irradiated lymphocytes and involved in acute toxicity. Click here for file

Additional file 7 **Functional Classification.** Late toxicity, 0 Gy. The table contains the functional classification in relation to biological process of genes regulated in un-irradiated lymphocytes and involved in late toxicity. Click here for file

Additional file 8 **Functional Classification.** Acute toxicity, 2 Gy. The table contains the functional classification in relation to biological process of genes regulated in irradiated lymphocytes and involved in acute toxicity. Click here for file
