# Supplementary material for: Constitutive gene expression profile segregates toxicity in locally advanced breast cancer patients treated with high-dose hyperfractionated radical radiotherapy
Source: Radiat Oncol. 2009 Jun 4;4:17. doi: 10.1186/1748-717X-4-17 (PMC2698866; doi:10.1186/1748-717X-4-17)
Supplement: Additional file 1 — Studies that have applied microarray analysis to compare gene expression profiles in patients with severe versus mild normal tissue damage after radiotherapy. Brief summary of studies related to radiotherapy and microarrays. The table includes the author's name and the year of publication, the cell type used the tumour type, some characteristics of the study and the most relevant findings. [file 1748-717X-4-17-S1.doc]

| **Additional file 1.** Studies that have applied microarray analysis to compare gene expression profiles in patients with severe versus mild normal tissue damage after radiotherapy. The table includes the author´s name and the year of publication, the cell type used, the tumor type, some characteristics of the study and the most relevant findings. | | | | | | |
| --- | --- | --- | --- | --- | --- | --- |
| ***Reference*** | ***Year*** | ***Type of Array*** | ***Cell Type*** | ***Tumor Type*** | ***Characteristics*** | ***Findings*** |
| Quarmby S. et al | 2002 | Cytokine array (268 probes) | Fibroblasts | Breast. 3 patients | Focused | 9 genes |
| Rieger KE. et al | 2004 | Affimetrix U95A_v2 | Lymphoblastoid cells | Different types.  14 cases (Breast, 4  patients) vs. 43 controls | Focused on acute toxicity *ex vivo* irradiation (4 Gy) | 24 genes |
| Svensson JP. et al | 2006 | Affimetrix HG-U133A | Lymphocytes | Prostate  21 cases vs. 17 controls | Focused. *ex vivo*  irradiation (0, 2 Gy) | 72 gene sets |
| Sonis S. et al | 2007 | Affimetrix HG-U133 plus 2.0 | Lymphocytes | Head and Neck  5 patients | Focused. Before vs.  after radiotherapy | 40 pathways |
| Rodningen OK. et al | 2008 | 15K cDNA microarray | Fibroblast cell lines | Breast. 31 Post-  mastectomy patients | Focused | 60 genes |
