# Supplementary material for: Constitutive gene expression profile segregates toxicity in locally advanced breast cancer patients treated with high-dose hyperfractionated radical radiotherapy
Source: Radiat Oncol. 2009 Jun 4;4:17. doi: 10.1186/1748-717X-4-17 (PMC2698866; doi:10.1186/1748-717X-4-17)
Supplement: Additional file 5 — Canonical pathways that were significantly modulated in the different set of genes. Pathways modulated and related to acute and late toxicity, 0 and 2 Gy. Pathway name, p-value, gene name and GeneBank accession number were included. [file 1748-717X-4-17-S5.doc]

| **Additional file 5.** Canonical pathways that were significantly modulated in the different set of genes, related to acute and late toxicity, 0 and 2 Gy. Pathway name, p-value, gene name and GeneBank accession number (in brackets) were included. Pathway-Express Onto-tool was used. No gene set was generated in late toxicity, 2Gy. | | | | | | |
| --- | --- | --- | --- | --- | --- | --- |
|  | ***Pathway Name*** | ***p- value*** | ***Genes Involved*** | ***Pathway Name*** | ***p-value*** | ***Genes Involved*** |
| ***0 Gy. 20 genes.*** | | | | ***2 Gy. 29 genes.*** | | |
| ***Acute Toxicity*** | Regulation of autophagy | 0.0003 | GABARAP [BM803698] | Phosphatidylinositol signaling system | 0.001 | PIP5K1C [U78575] |
|  | Vibrio cholerae infection | 0.0005 | SEC61G [AF054184] | TGF-beta signaling pathway | 0.0011 | SMAD6 [035528] |
|  | Protein export | 0.0001 | OXA1L [X80695] | Regulation of actin cytoskeleton | 0.0024 | PIP5K1C [U78575] |
|  | Phosphatidylinositol signaling system | 0.0008 | PIP5K1C [AB011161] | Cell cycle | 0.0014 | ORC5L [U92538] |
|  | Focal adhesion | 0.0018 | PIP5K1C [AB011161] |  |  |  |
|  | Regulation of actin cytoskeleton | 0.0019 | PIP5K1C [AB011161] |  |  |  |
| ***0 Gy. 26 genes.*** | | | | ***2 Gy.*** | | |
| ***Late Toxicity*** | Regulation of actin cytoskeleton | 0.0002 | PAK1 [AK055228]  GSN [AK125819] | *No hierarchical cluster constructed* | | |
|  | MAPK signaling pathway | 0.0003 | PAK1 [AK055228]  ECSIT [AF243044] |  |  |  |
|  | Epithelial cell signaling in Helicobacter pylori infection | 0.0007 | PAK1 [AK055228] |  |  |  |
|  | ErbB signaling pathway | 0.001 | PAK1 [AK055228] |  |  |  |
|  | Renal cell carcinoma | 0.0008 | PAK1 [AK055228] |  |  |  |
|  | Natural killer cell mediated cytotoxicity | 0.0012 | PAK1 [AK055228] |  |  |  |
|  | T cell receptor signaling pathway | 0.0009 | PAK1 [AK055228] |  |  |  |
|  | Axon guidance | 0.0013 | PAK1 [AK055228] |  |  |  |
|  | Focal adhesion | 0.0019 | PAK1 [AK055228] |  |  |  |
